# Factors Affecting Care Workers’ Coping Abilities in Emergencies to the Korean Elderly

**DOI:** 10.3390/ijerph16162946

**Published:** 2019-08-16

**Authors:** Soon-Ok Kim, JaeLan Shim

**Affiliations:** 1 Department of Nursing, Shinhan University, Uijeongbu 21936, Korea; 2 College of Medicine, Department of Nursing, Dongguk University, Gyeongju 38066, Korea

**Keywords:** elderly patients, emergencies, care workers, coping ability

## Abstract

This study provides basic data for enhancing coping abilities in emergencies concerning direct long-term care (LTC) workers, which is necessary for providing safe care for elderly patients living in facilities and at home. A survey was conducted including 327 care workers who officially qualified as long-term providers for elderly patients through elderly care facilities and a domiciliary service center. The majority (91.4%) of the care workers surveyed experienced an emergency, but of them, only 36.4% performed first aid and 56.8% failed to perform first aid, for which the emergency was reported to nurses. The average score regarding first aid knowledge was 8.40 out of 21, and the mean scores for the subtopics of basic life support and general first aid were low (3.56 out of 7 and 5.84 out of 14, respectively). Nearly three-quarters (72.5%) responded that they were unaware of emergency coping methods, and the score for coping abilities in emergencies was also low (52.93 out of 100). The results indicate that factors affecting coping abilities in emergencies were related to the size of the workplace and first aid experience. We propose the development and implementation of an emergency coping training program focusing on case studies for direct LTC workers.

## 1. Introduction

### Study Background 

According to a 2017 Report by the United Nations, the population of older persons aged 80 and over is predicted to increase more than three times, from 126 million in 2015 to 425 million by 2050; accordingly, it is urgent that measures be set up for elderly care [1]. South Korea is rapidly becoming an aging society, at a rate that no other country in the world has ever experienced. The country had already become an aged society in 2018 and is projected to become a super-aged society, with the elderly population making up 41% of its total population by 2060 [2]. One of the nation’s solutions to this social problem is the Long-Term Care Insurance (LTCI) scheme for older persons, introduced in July 2008, which provides older adults who have difficulty performing daily living activities on their own owing to dementia, geriatric diseases, and other conditions with long-term care services, including housework and physical activity support [3]. 

Aging causes many health issues for the elderly, and the incidence of chronic degenerative diseases is high. Thus, they tend to develop comorbid conditions, for which the causes are unclear and ambiguous and symptoms are complex and atypical. Older adults are also vulnerable to disturbances of consciousness due to the fact that they take various medications and suffer from cognitive disorders, including dementia [4,5,6]. As a result, older adults may face an emergency at any time, and with age, they are likely to need much more emergency care [7,8,9]. Those eligible for LCTI for older persons include patients with dementia, Parkinson’s disease, cerebrovascular disease, or degenerative diseases, so they are exposed to a much higher risk of mortality due to caused asphyxia and aspiration in an emergency. In fact, 40.7% of the staff working at elderly care facilities have experienced geriatric emergency accidents at least once in the past three months [10], and over 75% of all medical emergencies have been reported to occur at homes. Therefore, when providing services either in facilities or at homes, the risks of an emergency involving older persons are high [11]. To minimize damage from an emergency and an unexpected accident due to the worsening of disease in older patients, the caregivers need to be capable of responding well to emergencies by identifying potential emergencies that may occur with elderly patients; thus, making a quick assessment of the situation, as well as being able to call for emergency assistance when an emergency occurs to ensure that the first person to discover the situation can respond within the golden time so as to improve the survival rate of the elderly [12], is critical.

Direct long-term care (LTC) workers comprise a new type of caregiving workforce that emerged with the introduction of the LTCI scheme. They have been trained under a national certification system since February 2008. As of April 2018, there were approximately 1,516,880 certified elderly direct LTC workers, and there is no limit to the degree of medical care. If a care worker passes 240 hours of theoretical and practical training and passes the national examination at the National Health Examination Institute, a certificate is awarded. Employment is supported voluntarily at elderly care facilities and home care centers, and it is only necessary for the care worker to pass an interview with the facility director. According to the Elderly Welfare Act, the care worker provides facilities and home care services through the Elderly Care Facilities and the Home Care Center.

Institutional services provide assistance with physical activities and education to help maintain and improve mental and physical functions, while domiciliary care services include basic nursing services such as help with nutrition, toilet needs, personal hygiene, and mobility [3]. This shows that direct LTC workers are experts who provide care services for older adults around the clock and remain close to them, so they are likely to be the first to discover abnormal signs in the elderly [13]. Direct LTC workers can make a great difference in the survival and prognosis of the elderly because their response to an emergency may determine the life or death of an elderly person and shorten their recovery time [14]. However, as direct LTC workers are not medical service providers, they cannot perform direct emergency care. Thus, care workers need to be equipped with specialized skills and knowledge of geriatric emergencies so that they can play their role as “first responder” by recognizing an emergency and calling emergency services, such as medical personnel and EMTs (emergency medical technicians) [15,16]. 

Since 2014, education that direct LTC workers currently receive about emergencies has amounted to no more than a total of ten hours, consisting of a four-hour theory class and a six-hour practice session during the certification course; starting in 2016, the National Health Insurance Service has additionally offered and a total of three hours of training consisting of a two-hour theory class and a one-hour practical exposure to older patients. These courses are mostly focused on theoretical knowledge, suggesting that the training hours are far from sufficient to train direct LTC workers to properly respond to emergencies they frequently face when providing both domiciliary and institutional services. Thus, as no guidelines or legal standards concerning training for direct LTC workers in an emergency exist, it is imperative to introduce systematic training programs that educate direct LTC workers to make sound judgments regarding potential emergencies and to respond effectively. However, most studies on care worker emergencies in Korea after the institutionalization of long-term care insurance for the elderly have only been concerned with basic life support (BLS) [17,18,19,20] and emergency education that combines BLS and general first aid [21]; there is also little research on matters such as how direct LTC workers perceive emergencies of the elderly and how they cope with these emergencies. Therefore, it is necessary to identify the care workers’ level of emergency awareness, as they provide frontline care for the health of the elderly, as well as the factors affecting their coping abilities in emergencies under current circumstances wherein the advent of an aging society is accelerating and the incidence of emergencies concerning long-term care beneficiaries is continuously increasing. Through such identification, this study aims to provide results that can be utilized as basic data for the development of a training program that can enhance their coping abilities in emergencies, which is a core competency for direct LTC workers.

## 2. Materials and Methods 

### 2.1. Research Design and Procedure 

This study employed a descriptive survey design aimed to identify the level of emergency coping ability possessed by direct LTC workers and to investigate factors affecting the coping abilities of direct LTC workers in emergencies. 

The study participants included direct LTC workers who provide care services for elderly patients through elderly care facilities and domiciliary service centers. The number of participants was calculated using the G*power 3.1 program. For regression analysis, 172 participants were needed in total, using a significance level α = 0.05, power of 0.95, effect size of 0.15, and 10 predictive variables. We aimed for a total of 350 research questionnaires to be distributed in consideration of the dropout rate, and 327 research questionnaires were used for the final analysis; we excluded 23 research questionnaires that had incomplete responses.

The data collection was conducted from February 1 to March 5, 2019, in 10 elderly care facilities and eight home care centers. The researcher sent a formal letter to the Korean Elderly Welfare Facilities Association and Korean Association of Community Care for the Elderly, and before collecting data, the researchers obtained consent forms from the participants.

The researcher then directly visited the facilities and center, explained the purpose of the study to the supervisors of the institutions and obtained permission, after which the survey was conducted. The researcher directly explained to caregivers the necessity, purpose, and contents of the study and obtained a consent form for participation in the research; the data were then collected from those who agreed to participate in the research. The time required for the participants to complete the questionnaire was 25 to 30 minutes. See the Appendix A for the questionnaire used in the study.

#### Measurements

(1) Experience of emergencies and first aid knowledge

In this study, measurements that had been amended and supplemented by Kim and Kim [21] were used. In their survey of direct LTC workers, they modified 25 items that Kim and Lee [22] had designed to measure the experience of emergencies and first aid knowledge. In their study, scales for the experience of emergencies consisted of seven items, and scales for first aid knowledge included 18 items on BLS (eight items) and general first aid (10 items). This study added three more items to general emergencies (hypotension, abdominal pain, and drug or alcohol intoxication, which are frequent occurrences in elderly care facilities and domiciliary service centers). As a result, a total of 31 items were developed and used to measure the experience of emergencies (10 items) and first aid knowledge (21 items) including BLS (seven items) and general first aid knowledge (14 items). Amended measurements were used after the content validity index had been validated by a group of experts consisting of three nursing professors, two managers at elderly care facilities, and two directors of domiciliary care centers, with a content validity index of 0.82. 

● Experience of emergencies

Scales for the experience of emergencies consisted of a total of 10 items regarding these subjects: emergencies that direct LTC workers experienced at work in the past year and the time when they experienced them; frequency of each emergency; reporting system of emergencies; awareness of emergencies; why they were able or unable to provide first aid; what kind of first aid they provided; emergency situation coping method; and emergency response. The Cronbach’s α for Kim and Lee [22] was 0.84, Kim and Kim [21] 0.91, and 0.81 for this study.

● First aid knowledge

Scales for first aid knowledge consisted of a total of 21 items and were categorized into two parts: BLS and general first aid knowledge. The subscale of BLS contained a total of seven items regarding these subjects: time of a 119 call; open airway method; time of resuscitating the heart; ratio between compression and ventilation; number of chest compressions; compression depth and location of chest compression; and emergency response after a 119 call. The subscale of general first aid knowledge contained a total of 14 items regarding these subjects: decreased level of consciousness; airway obstruction; dyspnea; wound; trauma; sprain; syncope; hypoglycemia; stroke; head injury; convulsion; intoxication; epistaxis; and burn. The items on first aid knowledge were multiple-choice with four response options, and each correct answer scored one point, with a maximum score of 21. Higher scores indicate a higher level of first aid knowledge. The Cronbach’s α for Kim and Lee [22] was 0.87, Kim and Kim [21] 0.76, and 0.86 for this study.

(2) Care workers’ abilities to cope with emergencies

Care workers’ abilities to cope with emergencies refers to self-confidence in a situation wherein care workers encounter sudden changes (e.g., cardiac arrest) in the condition of a patient with traumatic or nontraumatic injury. To identify workers’ competence, measurements amended and supplemented by Kim Soo-Yeon et al. [23] and Kim and Kim [21] were used in this study. Kim Soo-Yeon et al. [23] modified measurements developed by Hwang et al. [24] in order to study the coping competence of care workers at a domiciliary service center in emergencies. Scales for coping competence consisted of a total of 20 items on a five-point Likert scale, and thus, the total score ranged from 20 to 100. A higher score indicates a higher level of self-confidence in emergencies. The Cronbach’s α for Hwang et. al [24] was 0.96, Kim Soo-Yeon et al. [23] 0.95, and 0.81 for this study. See the Appendix A for the questionnaire used in the study.

### 2.2. Ethical Considerations

The study was approved by the IRB of Shinhan University. (IRB No: SHIRB-201806-HR-085-02).

### 2.3. Statistical Analysis

Statistical analysis was performed using the SPSS for Windows version 22.0 (IBM, United States), and the statistical significance level was set as α = 0.05.

The participants’ general characteristics, experience of emergency, first aid knowledge, and coping abilities in emergency were analyzed by *χ*^2^ test, independent *t*-test, and one-way ANOVA and labeled according to frequency, percentage, mean, and standard deviation.

The participants’ general characteristics and differences in experiences of emergencies, first aid knowledge, and coping abilities during the emergency were analyzed by *χ*^2^ test, independent *t*-test, and one-way ANOVA. In case of statistical significance, a Scheffe test was used as the post-hoc test.

Correlations between emergency experience, first aid knowledge, and coping ability in emergencies were analyzed using Pearson’s correlation coefficients.

Factors affecting the ability to cope with emergencies were analyzed using hierarchical multiple regression analysis.

## 3. Results

### 3.1. Participants’ General Characteristics and Differences of Variables according to Those Characteristics 

Women accounted for 94.8% of the sample. Those under the age of 49 made up 11.9%; from the age of 50 to 59, 47.4%; and more than 60, 40.7% of the total. As for education level, high school graduates made up the largest category at 60.9%. Among these, 55.7% worked at elderly care facilities, while 44.3% worked at domiciliary care centers. Career duration as a care worker for one to four years was represented by 43.7% of the population and five to nine years by 32.4%. The emergency education cycle represented most frequently in the sample was three- to six-month intervals, accounting for 76.6% (Table 1).

Table 1 shows the differences in experience of emergencies, first aid knowledge, and coping ability in emergencies according to general characteristics. Differences in emergency experiences were statistically significant by age (*χ*^2^ = 7.49, *p* = 0.022), indicating that the age group under 49 had more experience with emergencies than the other age groups. With regard to experience by workplace, in addition, direct LTC workers working at elderly care facilities had significantly more emergency experience than those working at domiciliary care centers (*χ*^2^ = 21.24, *p* < 0.001). In addition, direct LTC workers trained at three to six-month intervals were found to have considerably more emergency experience than those trained every year or every other year (*χ*^2^ = 9.75, *p* = 0.012). However, there was no difference in emergency experience based on direct LTC workers’ gender, career, or education level.

For the analysis, first aid knowledge was divided into BLS and general first aid. First, there was no statistically significant difference in BLS by gender, age, education, workplace, working period, or education cycle. For general first aid, there was a significant difference according to gender (*t* = 5.65, *p* = 0.018), such that female workers had significantly more knowledge about general first aid than their male counterparts. Additionally, in terms of education, a statistically significant difference was seen (*t* = 9.17, *p* < 0.001) such that direct LTC workers with university or higher education had higher first aid knowledge scores. The general first aid knowledge of direct LTC workers in facilities was also significantly higher than that of those working at domiciliary service centers (*t* = 6.05, *p* < 0.001). There were no significant differences according to age, working experience, or emergency education cycle. For coping ability in emergencies, different workplaces showed significant differences, and facility workers showed a significantly higher ability to manage emergencies than those working at domiciliary centers (*t* = 5.74, *p* < 0.001). There was no corresponding significant difference according to gender, age, education, work experience, or emergency education cycle (Table 1).

### 3.2. Experience in Emergencies, First Aid Knowledge, and Emergency Coping Abilities of Care Workers

A total of 91.4% of the respondents had experienced emergency patients or experienced emergencies during the past working year, and 43.4% of them reported that the time period of experiencing the emergency was “regardless of day or night.” For how an emergency was discovered, change in external appearance was ranked at the top with 78.5%, and when an emergency happened, reporting to a nurse occurred 29.7% of the time and to an office (facility manager) 23.3% of the time (Table 2).

In all, 36.4% of the respondents had experience with first aid treatment; regarding a reason to conduct first aid, 68.1% of the subjects answered, “life seemed to be in a critical condition.” The reason given for not performing first aid was “not confident in first aid treatment,” at 38.0%; and “because emergency medical personnel had already performed first aid treatment,” at 19.2%. First aid knowledge was scored at 8.40 ± 3.07 points out of 21 points, BLS at 2.56 ± 1.36 points out of 7 points, and general first aid at 5.84 ± 2.36 points out of 7 points. For first aid methods, helping patients take medicine was the highest, with 71.9%, followed by vital signs, with 69.7%, and blood glucose tests, with 63.6% (Table 2).

For emergency coping abilities, 37.6% of the respondents answered that they were not confident because they did not know exactly how to cope with the situation. The score of coping ability in emergencies was low, at 52.93 ± 6.18 out of 100 points, 31.12 ± 3.31 out of 45 points for BLS, and 21.80 ± 2.87 points out of 55 points for general first aid (Table 2).

### 3.3. Correlations among Emergency Experience, First Aid Knowledge, and Coping Abilities in Emergencies

The results of examining the correlations between emergency experience, first aid knowledge (BLS and general first aid), and coping abilities in emergencies are shown in Table 3. Experience in emergencies positively correlated with first aid knowledge (*r* = 0.656, *p* < 0.001) and its subdomains, BLS (*r* = 0.593, *p* < 0.001) and general first aid knowledge (*r* = 0.772, *p* < 0.001). However, there was no significant correlation with coping abilities in emergencies. First aid knowledge showed a positive correlation with coping abilities in emergencies (*r* = 0.154, *p* < 0.001), indicating that the more first aid knowledge a respondent possesses, the higher their coping abilities in emergencies (Table 3).

### 3.4. Factors Affecting Coping Abilities in Emergencies

The results of the hierarchical multiple regression analysis on the factors affecting coping abilities in emergencies are shown in Table 4. In order to test the assumption of linear regression analysis, all variables were checked for normality and linearity, and the Dubin–Watson statistic was 2.03, which was close to the reference value of 2, so there was no problem of autocorrelation. The variance inflation factor was confirmed to indicate no problem of multicollinearity, as the values of variables included in all models were not greater than 10.

In model 1, the general characteristics, age and education, type of workplace, facility size were applied after dummy variable treatment, and the regression model was found to be statistically significant (*F* = 24.41, *p* ≤ 0.001). The explanatory power of the first stage model was 20.1%. The statistically significant variable was a facility size of more than 10 people (*β* = 3.24, *p* < 0.001). In stage 2 (model 2), first aid knowledge and perception of the emergency were added together as variables after emergency experience was treated as a dummy variable, and the regression model was statistically significant (*F* = 26.76, *p* ≤ 0.001). The explanatory power of this model was 23.1%. In model 2, significant factors affecting coping ability in the emergency were a facility size of more than 10 people (*β* = 0.30, *p* < 0.001) and experience of first aid (*β* = 0.33, *p* < 0.001). The explanatory power of the final model (model 2) was 23.1%. Higher coping abilities in emergencies were related to larger workplaces and previous experience in emergency situations. 

## 4. Discussion

This study aimed to analyze emergencies experienced by direct LTC workers who provide long-term care services in elderly care facilities and domiciliary care centers, as well as the influencing factors on coping abilities in emergencies when elderly patients exhibit sudden health changes. The analysis was conducted to provide basic data for developing educational programs to enhance direct LTC workers’ coping abilities in emergencies and to contribute to improving the quality of long-term care services for elderly patients.

The results of this study show that over the last year, 91.4% of direct LTC workers experienced emergencies while caring for the elderly and 43.4% said they experienced them day and night, indicating that emergencies in their service fields happen frequently. However, the results of this study are different from those of previous studies. Kim Soo-Yeon et al. [23] conducted a survey over three months on 76.7% of elderly care facilities and 53.5% of domiciliary care centers. A study by Lee Jae-min [19] looked into the experiences of emergencies requiring the performance of basic life support, and found that 21.2% of workers experienced emergencies. The rate of emergency situation experiences in this study is higher than in other research because this study included all basic life support and general first aid, in addition to traditionally defined emergency situations, over the course of one year.

An additional examination of emergencies experienced by participants reveals that the highest percentage of emergencies, 49.5%, were related to difficulty in breathing, asphyxia, and loss of consciousness, followed by injury and fracture at 25.4%; this was similar to findings reported by prior studies [14] conducted on many long-term care service fields. Thus, this finding corroborates the concern that the elderly are likely to be exposed to emergencies such as asphyxia or aspiration [25,26]. When these emergencies occur, the care workers first report them to the nurse (29.7%) and then inform the facility manager or office.

When it was judged that an elderly patient was in critical condition, 36.4% of the direct LTC workers provided first aid; 68.1% of the first aid was given when the care worker sensed that the patient’s life was in danger and 27.7% was given when a care worker felt capable of giving first aid. On the other hand, 63.6% did not give first aid and 56.8% said that they were not confident in their capabilities and afraid that the patients’ condition may worsen after giving first aid, indicating that they lacked confidence in their ability. Knowledge of first aid was low in general: results show scores of 8.40 out of 21 points in knowledge on first aid (an average score of 40.0 out of 100), 3.56 out of 7 points in BLS, and 5.84 out of 14 points in general first aid, showing a correct answer rate below 60%. This seems to be a result of insufficient training times: 10 hours for first aid training and 3 hours of a basic course for dementia. Seon-Ok Ahn [27] reported that the more chances for education there are, the higher the level of knowledge. In Korea, several previous studies [28] reported that the knowledge of emergency care is higher when participants have received emergency education. In a foreign study [29], it was emphasized that practitioner education and support for symptom management of emergencies in elderly care facilities should be strengthened for those who are in charge of the end stage in the elderly’s lives. Therefore, one policy recommendation is that the government should institutionalize education for care workers by providing compulsory education in a structured way [12].

In addition, subjects with a bachelor’s degree have a higher level of first aid knowledge, which is consistent with the results of earlier studies [17,19]. Therefore, it is necessary that first aid education be given taking into account the educational levels of direct LTC workers. 

Among direct LTC workers, 72.5% said they did not know how to cope with an emergency, and the results of the survey on coping abilities in emergencies yielded an average score of 52.93 out of 100 points, which was rather low. Among such coping abilities, general first aid was rated at 21.80 out of 55 points, or 39.6%, and BLS was rated at 31.12 out of 45 points; 68.8% of participants said they were confident in dealing with emergencies. These results may reflect the fact that most of the first aid training offered to direct LTC workers up to now has focused on BLS [21]. Therefore, education on general first aid as well as BLS should be offered so that direct LTC workers can properly understand various types of emergencies taking place in their service field and cope with them. In addition, the results of this study are consistent with the results of earlier studies [9,30], which reported that care workers with higher first aid knowledge are better at coping with emergencies. These care workers could improve their coping abilities in emergencies; their current expertise on first aid has been accumulated from different experiences of caring for the elderly along with theoretical knowledge of first aid. Hence, there is need to strengthen first aid education in the future.

Our study suggests that workers at elderly care facilities have better coping abilities in emergencies than do domiciliary care centers, and a regression model found that the type of workplace is a relevant factor. These results are consistent with those of Kim Soo-Yeon et al. [23]. The elderly can be divided into five levels based on their health condition or body dependency, and they are classified into facilities and households according to their grade status. The higher the rating, the lower the severity. The elderly in care facilities have first- to second-class severity, more severe than those in domiciliary care centers, and their ability to perform daily activities is relatively lower [31], so they are in need of specialized medical services. Therefore, direct LTC workers in elderly care facilities may have better coping ability in emergencies as they have gained knowledge of first aid through diverse experiences in performing first aid in these circumstances [32]. Park’s study [31] of the performance level of care workers’ duties in elderly care facilities and domiciliary care centers shows that in all four domains (physical, emotional, duties beyond normal duties, and first aid), direct LTC workers in elderly care facilities have a higher performance level than those in domiciliary care centers, confirming that there is a difference between these two workplaces. This study did not examine differences in coping abilities in emergencies between those who work in elderly care facilities and domiciliary care centers, so further research in this regard is needed.

In summary, the results of these studies indicate the need to enhance the coping abilities of direct LTC workers in an emergency so that such workers can quickly recognize and address medical emergencies in elderly patients in accordance with proper first aid procedures within the golden time, since such direct LTC workers are likely to be the first people to find older patients experiencing urgent medical conditions. As a policy recommendation, emergency training, including education on general first aid skills as well as BLS, should be provided to these workers, even though they are not medical professionals. It is also imperative that education programs be developed for direct LTC workers for making proper first aid decisions and handling emergencies without errors of judgment in actual settings. Additionally, considering that the average age of the care workers in this study is relatively high at 57.29, it is necessary to develop educational content using plain language rather than medical terminology in order to facilitate understanding. In this case, repeated short-term training courses can have a greater impact on the education of such care workers. Additionally, this study shows that workers at care facilities have more experience in handling emergencies, and hence have better skills, than those in domiciliary facilities. They have acquired first aid knowledge within a shorter period of time from their first-hand experience of various medical emergencies while spending longer hours with a higher number of elderly patients, whom they take care of simultaneously, than domiciliary care workers do. In this respect, it is necessary for domiciliary care workers to be provided with education on various emergencies to strengthen their coping abilities in actual healthcare settings.

Long-term care services for the elderly require continuous training for direct LTC workers [33]. However, countries, such as the Netherlands and Denmark, where a long-term care system was implemented earlier, there is still a lack of system for continuing job education [34]. It has been reported that the government is promoting a variety of policy systems, including education and training, as well as career development [35]. Although it has been ten years since the introduction of the long-term care insurance system in Korea, job training still consists of eight hours of job training and 60 hours of dementia education training beyond the typical 240 hours of training, and there is no systematic job training or maintenance education system. This is insufficient. Furthermore, in the United States, where long-term services for the elderly have been developed, education programs have been prepared to strengthen the professional and detailed emergency response capabilities by administering theoretical and clinical education regarding emergency medical care for “home health aides” [36]. 

Therefore, this study is meaningful in that it has been able to recognize the abilities, including coping abilities, of caregivers who are relatively new to Korea. In addition, based on the results of this study, case-based emergency education was developed and applied to build up knowledge about first aid treatment and to solve the discussed problems. This will serve as a basis for the establishment of an education system for each field of specialization. The results of this study can be used as basic data for the preparation of emergency guidelines, as well as guidelines for emergency treatment in the field of long-term care services. 

While the results of this study provide a number of meaningful insights, it is limited in the following aspects. Generalization of the results of this study requires careful consideration, as the participants in the study were selected through convenience sampling from workers at elderly care facilities and domiciliary centers in some regions. In addition, interpretation of the findings of this study should be based on a thorough examination, since this study measured research variables based on self-report surveys that may contain biases. In the future, further efforts are required to objectively evaluate the performance of direct LTC workers based on direct observation, for which systematic education strategies are a prerequisite.

## 5. Conclusions

In this study, we investigated in detail the emergencies experienced and the corresponding coping competence of direct LTC workers, the core workforce of the long-term care service, in a situation wherein the number of beneficiaries of long-term care service is rapidly increasing. The significance of this research lies in its potential contribution to an increase in the elderly survival rate and the improvement of long-term care service quality by enhancing the coping abilities of care workers in emergencies. Recommendations include improving the coping abilities of such care workers, ensuring survival of elderly patients in emergencies, safety management, and the development and implementation customized emergency education.

## Figures and Tables

**Table 1:** Participants’ general characteristics of and differences of variables according to those characteristics (*N* = 327).

Characteristics	Categories	*n*	Experience of Emergency Situation			Knowledge in Emergency Situation				Coping ability in Emergency Situation	
						BLS		General First Aid			
			Yes, n(%)	No, n(%)	*χ*^2^ (*p*)	Mean ± SD	*t* or *F* *(p)*	Mean ± SD	*t* or *F* (*p*) (Scheffe)	Mean ± *SD*	*t* or *F* (*p*) (Scheffe)
Gender	Male	17	17(100.0)	0(0.0)	1.68 (0.210)	2.18 ± 1.78	0.01 (0.991)	4.53 ± 2.85	5.65 (0.018)	50.99 ± 4.50	0.29 (0.770)
	Female	310	282(91.0)	28(9.0)		2.58 ± 1.34		5.92 ± 2.31		54.47 ± 5.42	
Age in years (57.29 ± 7.53)	Less than 49	39	38(97.4)	1(2.6)	7.49 (0.022)	2.18 ± 1.32	0.86 (0.414)	5.85 ± 2.82	1.84 (0.160 *)	52.41 ± 5.37	1.52 (0.221)
	50–59	155	146(94.2)	9(5.8)		2.85 ± 1.29		6.09 ± 2.20		53.46 ± 5.57	
	over 60	133	115(86.5)	18(13.5)		2.33 ± 1.40		5.56 ± 2.38		52.45 ± 4.96	
Education	Middle school or less ^a^	67	61(91.0)	6(9.0)	4.75 (0.73)	2.27 ± 1.34	1.47 (0.232)	5.04 ± 2.16	9.17 (<0.001 *) (a, b < c)	52.37 ± 5.07	0.70 (0.499)
	High school ^b^	199	178(89.4)	21(10.6)		2.65 ± 1.36		5.82 ± 2.31		53.20 ± 5.52	
	College or above ^c^	61	60(98.4)	1(1.6)		2.57 ± 1.38		6.79 ± 2.42		52.66 ± 4.90	
Workplace	Home	145	121(83.4)	24(16.6)	21.24 (<0.001)	2.46 ± 1.31	1.40(0.162)	5.18 ± 2.14	6.05 (<0.001)	50.99 ± 4.50	5.74 (< 0.001)
	Facility	182	178(97.8)	4(2.2)		2.69 ± 1.43		6.68 ± 2.36		54.47 ± 5.42	
Length of service	Less than 1 year	50	45(90.0)	5(10.0)	4.44 (0.205)	2.56 ± 1.50	1.50 (0.215)	5.76 ± 2.88	0.54 (0.650 *)	52.32 ± 4.74	1.96(0.075)
	1 to 4 years	143	127(88.8)	16(11.2)		2.45 ± 1.34		5.72 ± 2.32		52.90 ± 5.62	
	5 to 9 years	106	99(93.4)	7(6.6)		2.75 ± 1.42		6.08 ± 2.20		53.22 ± 5.03	
	Over 10 years	28	28(100.0)	0(0.0)		2.39 ± 0.99		5.71 ± 2.14.		53.07 ± 5.88	
Frequency of emergency education	Every 3–6 months	244	60(100.0)	0(0.0)	9.75 (0.012)	2.89 ± 1.48	1.38 (0.226)	6.35 ± 2.39	1.72 (0.121 *)	54.80 ± 5.41	0.71(0.545)
								6.11 ± 2.20		52.80 ± 5.37	
	every 1 year	76	67(89.3)	8(10.7)		2.57 ± 1.53		6.00 ± 2.32		52.20 ± 5.42	
	every 2 years	7	5(71.4)	2(28.6)		2.14 ± 0.90		6.29 ± 1.80		53.29 ± 5.85	

* by the one-way ANOVA (post-test Scheffe) for three or more groups. BLS: Basic Life Support.

**Table 2:** Emergency situation experience, first aid knowledge, and emergency coping abilities (*N* = 327).

Variables	Characteristics	Categories		*n* (%) or Mean ± *SD*
Experience of emergency situation	Emergency experience	Yes		299(91.4)
		No		28(8.6)
	Experience timing of emergency situation	Daytime		124(37.9)
		Night		40(12.2)
		Daytime, Night		163(49.8)
	Identification method for abnormal signals *	Change in appearance		256(78.5)
		Change in feelings of touch		216(66.3)
		Change in behavior		201(61.7)
		Change in responses		153(46.9)
	Report of emergency situation	Manager		76(23.2)
		Nurse		97(29.7)
		Emergency Medical Technician		62(19.0)
		Care worker		62(19.0)
		Nurse aide		30(9.2)
	Details of emergency situation	Difficulty breathing, asphyxia, loss of consciousness, Dysphagia, cardiac arrest, etc.		148(49.5)
		Fracture, sprains, trauma, etc.		76(25.4)
		Hyperglycemia (hypoglycemia), dementia-related mental behavior symptoms		54(18.1)
		Other, including abdominal pain, dehydration, bleeding, blood in stool, burns, etc.		21(7.0)
First aid knowledge	First aid experience	Yes		119(36.4)
		Reason for action	Life-threatening	81(68.1)
			First aid ability	33(27.7)
			Other	5(4.2)
		No		
		Reason for no action	No confidence	79(38.0)
			No equipment	21(10.1)
			Worries of making it worse	39(18.8)
			Causing legal problems	18(8.7)
			Medical professional should act	40(19.2)
			Other	11(5.3)
	Emergency situation coping methods	No knowledge at all		114(34.9)
		No accurate knowledge		123(37.6)
		Good knowledge		65(19.9)
		Other		25(7.6)
	First aid knowledge	Total		8.40 ± 3.07
		Basic life support		2.56 ± 1.36
		General first aid		5.84 ± 2.36
Coping ability in emergency situation	Emergency situation coping abilities	Total		52.93 ± 6.18
		Basic life support		31.12 ± 3.31
		General first aid		21.80 ± 2.87

* Multiple response.

**Table 3:** Intercorrelation coefficients among the measured variables (*N* = 327).

Category	Emergency Situation Experience	First Aid Knowledge (Total)	First Aid Knowledge		Coping Abilities in Emergency Situation
			*BLS Knowledge*	*General First Aid Knowledge*	
Emergency situation experience	1				
First aid knowledge	0.656 **	1			
*BLS knowledge*	0.593 **	0.131 *	1		
*General first aid knowledge*	0.772 **	0.151 **	0.318 **	1	
Coping abilities in emergency situation	0.051	0.154 **	0.016	0.013	1

** *p* < 0.001.

**Table 4:** Regression analysis for predictive factors of ability to cope with emergencies (*N* = 327).

Predictors	Model I			Model II		
	β (*P*)	95% CI		β (*P*)	95% CI	
		Lower	Upper		Lower	Upper
Age (Ref: <50 years)	0.01 (0.838)	−0.07–1.00		0.01 (0.865)	−0.07–1.00	
Education (Ref: <college)	−0.21 (0.773)	−1.60–1.20		−0.01 (0.863)	−1.54–1.30	
Workplace (Ref: home)	−0.89 (0.346)	−2.74–0.96		−0.10 (0.267)	−3.07–0.85	
Size of workplace (Ref: <10 people)	3.24 (0.001)	1.34–5.13		0.30 (0.001)	1.29–5.14	
First aid experience (Ref: No)				−0.04 (0.469)	−2.80–1.29	
Emergency knowledge score (BLS)				0.01 (0.860)	−0.39–0.46	
Emergency knowledge score (general first aid)				0.03 (0.666)	−0.21–0.32	
Experience of emergency situation (Ref: No)				0.33(0.001)	1.44–5.59	
*F*(*P*)	24.41(<0.001)			26.76(<0.001)		
*R* ^2^	0.214			0.257		
Adjusted *R*^2^	0.201			0.231		

Note. CI = Confidence Interval.

## Appendix A

**Ⅰ.** Questions about your experience with emergency situations and first aid experience

1. Have you ever witnessed an emergency or experienced an emergency during your workday?

① Yes
② No

2. If you have had an emergency, how many times have you experienced during the past year?

① 5 or less
② 6–10
③ 11–15
④ 16–20
⑤ 21 times

3. When did you experience the emergency situation of your elderly person?

① Day
② Night
③ Day or night
④ Dawn
⑤ Others

4. If you have ever witnessed an emergency patient, what was the patient?

① Difficulty breathing, asphyxia, loss of consciousness, dysphagia, cardiac arrest, etc.
② Fracture, sprains, trauma, etc.
③ Hyperglycemia, hypoglycemia, dementia-related mental behavior symptoms
④ Hypertension, Hypotension
⑤ Other, including abdominal pain, dehydration, bleeding, blood in stool, burns, etc.

5. Who will be the first to know if an elderly employee is aware that an emergency has occurred?

① Office (Facility)
② Nurse
③ Nursing assistant
④ Care giver
⑤ Emergency medical technician

6. How do you identify your emergency situation? All applicable items Please check.

① Change of appearance: skin redness, pale pus, cold sweat, etc.
② Changes in touch: edema, skin temperature, etc.
③ Change of behavior: Asphyxiation, change of physical condition, different behavior from usual
④ Change of reaction: change of vital signs, abnormal breathing, change of consciousness, no response to stimulation
⑤ No special method

**Ⅱ.** Questions about first aid experience. Please mark “✓” in the corresponding box.

1. Do you have any first aid experience with your elderly person during your last year?

① Yes (Only answer 1-1)
② No (Please answer only 1-2 times)

1-1. What is the reason for the first aid treatment in Step 1 above?

① I think life may be dangerous
② I think I can do first aid
③ The paramedics arrive late
④ Care worker should conduct
⑤ Others

1-2. What is the biggest reason if you did not do first aid in step 7 above?

① I am not confident in first aid treatment
② I do not have first aid equipment
③ When I was first aid, my elderly condition could be worse
④ When I was first aid, I thought it could be a legal matter
⑤ Emergency treatment must be carried out by an emergency medical worker

2. What do you think about emergency treatment in case of an emergency?

① I do not know how to deal with it
② I know a little how to cope but it is difficult to cope
③ I know how to cope, but I do not have confidence because I do not know exactly
④ I know how to deal with it, and I have no problem to deal with it
⑤ Other

**Ⅲ.** Questions about first aid knowledge. Please “v” to the appropriate number “as you know it”.

**1. Basic life support**

(1) If you find an elderly person who has fallen while you are alone and you have no movement at all, should?

① Immediately after confirming that there is no consciousness
② After lifting his head and lifting his chin
③ After blowing in the artificial respiration
④ After 2 minutes of CPR

(2) The old man was unconscious and there was no movement and he asked 119 for help. What first aid should we do next?

① Raise your head back and raise your chin
② Breathe in your breath
③ Press your chest with both hands
④ Fix the neck bone

(3) When you perform cardiopulmonary resuscitation on a cardiac arrest patient, do you think it is likely to be resuscitated when you start?

① Within 4 minutes after cardiac arrest
② Within 8 minutes after cardiac arrest
③ Within 12 minutes after cardiac arrest
④ Within 16 minutes after cardiac arrest

(4) Where should I press my chest when a heart attack is suspected?

① Left chest
② Right chest
③ Upper half of middle half of chest
④ Middle of chest between both nipples

(5) How many times do you have chest compressions and artificial respiration when you are doing cardiopulmonary resuscitation alone to a suspected heart attack?

① 3 times: 1 time
② 5 times: 1 time
③ 15 times: 2 times
④ 30 times: 2 times

(6) Which of the following is true for the depth of chest compressions when performing chest compressions to the elderly?

① minimum 2cm
② minimum 3cm
③ minimum 4cm
④ minimum 5cm

(7) What is the right thing to do right after giving an electric shock to an automatic defibrillator?

① Perform artificial respiration
② Watch whether the patient is responding
③ Press the chest
④ Check the pulse

**2. General First Aid**

(1) The elderly person who suffers from respiratory disease is breathing, breathing badly and changing lips. What should I do?

① Lay down comfortably
② Take a comfortable position such as sitting and breathing
③ Let’s lay down
④ Take a position to lower your head and raise your legs

(2) It is difficult for the elderly who have food to be caught in the middle of the meal to breathe, but what should I do if I have consciousness to induce a cough, but no food?

① Implement the Himiri Law
② I tug my back strongly
③ I knock my chest
④ Feed water

(3) What should I do when elderly suddenly feels angry and angina such as difficulty in breathing, cold sweating, cyanosis, complaining of chest pain like tightening and tightening?

① Call 119 right away
② Pain relief to relieve chest pain
③ Rest comfortably
④ The head is lowered and the legs are raised

(4) I want to bleed to the elderly person who is wounded and sheds blood. What should I do?

① Leave it alone
② Cover the wound with a clean cloth and press it with your hand
③ Wrap the wound with a warm towel
④ Wash the blood with water

(5) How should I cope with an elderly person who is suspected of hypotensive shock?

① Provide food
② Get up and do activities
③ Lift your legs about 20–30 cm
④ Raise your head about 20–30 cm and rest easy

(6) The elderly grabbing his abdomen and suffering from severe abdominal pain. What is the correct way to do this?

① Provide leprosy because constipation is suspected
② It is a temporary phenomenon and provides analgesics
③ Rub your ship and rest assured that it will be okay in a little while
④ Do not provide food, accurately identify the pain site and report to the manager

(7) If the elderly person suddenly falls unconscious and falls down and faints, what is the correct way to cope?

① Measure your blood pressure and raise your legs
② Provide cold water
③ Take sleep
④ Provide food

(8) The elderly suddenly stiffen the whole body and cramp in the form of moving arms and legs. What should I do?

① Hold your arms and legs to avoid cramps
② Check for dangerous objects around and remove
③ Pass the towel between the patient’s teeth
④ Hold your hand

(9) After he slipped in the bathroom, he said that his right ankle was bruised and swollen and could not move. How Should I?

① Grind it
② Give it a splint so it will not move
③ Put on warm fomentation
④ Lower the ankle below the heart

(10) The elderly who are caring have diabetes and are taking diabetes medication. The elderly suddenly sweat cold. It is in a state of consciousness, although it is hanging down and without energy. What is the correct approach?

① After measuring blood sugar, feed juice
② Report to 119 and wait until paramedics come
③ Feed warm water
④ I am tired and therefore take sleep

(11) Suddenly the face was red, and the body temperature was measured by appealing to chills, and a high fever of 38.5 degrees appeared. How to cope?

① feed a lot of water
② measure the body temperature, then feed the fever
③ Take off your old clothes and wipe them with a lukewarm water towel
④ Because you may have quit, try digestion agent

(12) The elderly shed a nosebleed. How should we cope?

① After tightening your nose, pressure your nose
② Turn your head back and press the dorsum nasi
③ pressure the nose while warming the panting
④ Lean your head slightly forward and pressure your nose

(13) The elderly person was burned with hot water. What should I do?

① Soak in cold water
② Remove the clothes attached to the skin as soon as possible
③ Burned arms and legs are dropped
④ Blisters are blown out

(14) Dementia When the old man drinks milk as a kitchen detergent (if he/she misuses the harmful substance) How to deal with emergency?

① Use your finger to vomit
② Drink water
③ Feed the milk, neutralize it, and bring it to the hospital
④ Contact 119 to tell the type of detergent and follow the instructions

**Ⅳ.** What do you think is the ability to cope when an emergency occurs?

Please “v” to the appropriate number “as you know it”.

classification	content	Never	No	Usually	Good	Extremely Good
*BLS*	1. You can check whether or not you have changed your consciousness.					
	2. You can check whether the unconscious master is breathing.					
	3. You can maintain the prayer of unconscious master.					
	4. You can distinguish whether there is a pulse or not by touching the neck artery of an unconscious master.					
	5. I can choose the chest pressure position for the non-pulse person.					
	6. Know your first aid kit and AED (automatic defibrillator) location.					
	7. I can activate the AED (Automatic Defibrillator).					
	8. When using AED (Automatic Defibrillator), I know about pad attachment area.					
	9. When using the AED (Automatic Defibrillator), you can re-confirm that someone is away from the subject before pressing the Shock button.					
*General first aid*	10. You can distinguish the elderly from the dangerous situation (whether it is safe).					
	11. You can choose your chest pressure position for the elderly without motion					
	12. Follow chest compressions depth and speed, and chest compressions can be performed.					
	13. When performing artificial respiration, you can open your airway by lifting your head and lifting your jaw.					
	14. You can breathe for one second to get your chest up when you apply artificial respiration.					
	15. Abdominal thrust can be performed when foreign body is obstructing the airway.					
	16. It is possible to fix the part where the fracture is suspected or not by moving or falling.					
	17. Hemorrhagic bleeding can be done by direct compression of the bleeding site.					
	18. Emergency treatment can be given to a burned elderly person.					
	19. You can take an attitude that helps the elderly who are suspected of shock.					
	20. If you find an unconscious master, you can immediately request a rescue at 119					

**Ⅴ.** This is a general question. ✓ Please mark or fill in the details.

1. What is your gender?

① Man
② Woman

2. What is your age? (   )

3. What is your final academic background?

① Graduated elementary school
② Graduated from junior high school
③ Graduated from high school
④ Graduated from university

4. What career have you been working as a care worker?

① Less than 1 year
② More than 1 year to less than 3 years
③ More than 3 years to less than 5 years
④ More than 5 years ~ Less than 10 years
⑤ More than 10 years

5. What is your current workplace?

① Elderly care facilities

a. Elderly Care Facilities 10 or fewer (   )

b. Elderly care facilities: 10–29 (   ), 30–49 (   ), 50–99 (   ), more than 100 (   )

② Home care center

a. Visit care   b. Day/night protection   c. Short-term protection   d. Visit Bath

6. How often did you receive first aid training?

① Every 3 months
② Every 6 months
③ Every year
④ Every 2 years
